# Part-time and full-time medical specialists, are there differences in allocation of time?

**DOI:** 10.1186/1472-6963-6-26

**Published:** 2006-03-03

**Authors:** Judith D de Jong, Phil Heiligers, Peter P Groenewegen, Lammert Hingstman

**Affiliations:** 1 NIVEL - Netherlands institute for health services research, PO Box 1568, 3500 BN Utrecht, The Netherlands; 2 Utrecht University, Department of Social Sciences, Utrecht, The Netherlands

## Abstract

**Background:**

An increasing number of medical specialists prefer to work part-time. This development can be found worldwide. Problems to be faced in the realization of part-time work in medicine include the division of night and weekend shifts, as well as communication between physicians and continuity of care. People tend to think that physicians working part-time are less devoted to their work, implying that full-time physicians complete a greater number of tasks. The central question in this article is whether part-time medical specialists allocate their time differently to their tasks than full-time medical specialists.

**Methods:**

A questionnaire was sent by mail to all internists (N = 817), surgeons (N = 693) and radiologists (N = 621) working in general hospitals in the Netherlands. Questions were asked about the actual situation, such as hours worked and night and weekend shifts. The response was 53% (n = 411) for internists, 52% (n = 359) for surgeons, and 36% (n = 213) for radiologists. Due to non-response on specific questions there were 367 internists, 316 surgeons, and 71 radiologists included in the analyses. Multilevel analyses were used to analyze the data.

**Results:**

Part-time medical specialists do not spend proportionally more time on direct patient care. With respect to night and weekend shifts, part-time medical specialists account for proportionally more or an equal share of these shifts. The number of hours worked per FTE is higher for part-time than for full-time medical specialists, although this difference is only significant for surgeons.

**Conclusion:**

In general, part-time medical specialists do their share of the job. However, we focussed on input only. Besides input, output like the numbers of services provided deserves attention as well. The trend in medicine towards more part-time work has an important consequence: more medical specialists are needed to get the work done. Therefore, a greater number of medical specialists have to be trained. Part-time work is not only a female concern; there are also (international) trends for male medical specialists that show a decline in the number of hours worked. This indicates an overall change in attitudes towards the number of hours medical specialists should work.

## Background

As more and more medical specialists prefer to work part-time [e.g. [1,2]], the question arises as to the consequences of this development for the tasks carried out by medical specialists. The trend of an increasing number of part-time medical specialists is expected to continue, since it is predominantly women who work part-time, and the percentage of female medical specialists is increasing [3-5]. In the Netherlands 50% to 60% of all medical students are female [6]. Growing numbers of female medical students can be found in other countries as well, e.g. the UK, Australia, New Zealand, Canada and the US, with women making up nearly half of the medical student population [7-12].

Problems to be faced in the realization of part-time work in medicine include the distribution of night and weekend shifts, communication between physicians and continuity of care. People tend to think that physicians who work part-time are less devoted to their work, implying that more tasks are completed by full-time physicians [3,13,14]. However, results from several studies give no clear answer to the question as to whether part-time employees are less dedicated to their work or not [15]. A distinction can be made between 'in-role' and 'extra-role' behaviour; 'in-role' behaviour relates to the core task of medical specialists, and 'extra-role' behaviour refers to all tasks which are not formal tasks of medical specialists [16].

In this article we will consider all tasks that do not belong to the core business of medical specialists, although they are formal tasks, as 'extra-role'. This is done to distinguish differences in the importance of tasks. Patient care can be considered 'in-role' behaviour, while other tasks like administration and management can be considered 'extra-role' behaviour. Part-time physicians might exhibit less 'extra-role' behaviour. Furthermore, when working with part-time physicians there are more occasions when work needs to be transferred. As a result, more time may be spent on inter-professional communication.

The central question in this article is whether part-time medical specialists allocate their time differently over their tasks than full-time specialists. We will examine whether specialists working part-time devote more time to patient care ('in-role' behaviour) in proportion to other tasks ('extra-role' behaviour), such as administration, and whether full-time physicians complete proportionally more night and weekend shifts. Additionally, we will examine the number of hours worked per Fulltime equivalent (FTE), for both part-time and full-time medical specialists. Before answering these questions we will explain the importance of this study, and formulate expectations.

What are reasons for studying the potential organizational difficulties related to part-time work? In general, part-time work is to a large extent gendered; most part-time workers are women [9,17]. Women are trying to find a balance between career and family life and part-time work has become an instrument to realise this balance [18-20]. For instance, Dutch female employees prefer to continue their jobs when they have young children, but the majority will achieve this only if they can reduce working hours, usually by half [17]. In order to make the labour market accessible for women at all stages of their working life the possibility of part-time work is important. When we focus on the health care sector, this is not only important for individual women, but also for the health care sector at large. Women might choose not to work as medical specialists when there is no option for part-time work, and this could result in medical workforce shortages. Furthermore, medical specialists who resign, for example when they have young children, will lose skills, making it more difficult to re-enter the labour market. For highly skilled women, like female medical specialists, the opportunity cost of becoming a housewife after many years of training and having a position as a medical specialist is high. This brings us to an argument put forward by those opposing part-time work among medical specialists: their education was expensive and therefore they should work full-time. Another argument against part-time work is that when there are part-time medical specialists more time is needed for inter-professional communication, and this takes place at the expense of other tasks.

Working part-time will probably have an effect on what is or can be done; it is most likely that not all tasks medical specialists perform are considered equally important. *We expect that part-time medical specialists devote more time to the core business of their work, that they perform proportionally more 'in-role' behaviour (1)*. This means that they will spend proportionally more time on direct patient care, and less on other tasks. When working part-time there are more moments of transfer. Therefore, *we expect part-time medical specialists to spend proportionally more time on inter-professional communication (2)*.

Night and weekend shifts, although part of a medical specialist's job, go beyond regular working hours. Part-time medical specialists are more involved in non-work-related responsibilities in comparison with full-time medical specialists. This could make part-time medical specialists less flexible, and therefore influence their availability in acute tasks, as well as in night and weekend shifts. *We expect that part-time medical specialists complete proportionally fewer night and weekend shifts than full-time medical specialists (3)*.

If part-time medical specialists are less efficient than full-time medical specialists, this may be compensated in the number of hours worked per FTE. Possibly, part-time medical specialists work more hours per FTE in order to do their part of the job. *We expect the number of hours worked per FTE to be higher for part-time than for full-time medical specialists (4)*.

Summarizing, we have four expectations. We expect part-time medical specialists to:

1) devote proportionally more time to patient care, the core business of their work;

2) spend proportionally more time on inter-professional communication;

3) complete proportionally fewer night and weekend shifts than full-time medical specialists;

4) have a higher number of hours worked per FTE.

## Methods

### Data

A questionnaire was sent by mail to all internists (N = 817), surgeons (N = 693) and radiologists (N = 621) working in general hospitals in the Netherlands. These different specialties were chosen on the basis of differences in procedures and organization of work, connected with characteristics of tasks, patient contacts and cooperation with other disciplines.

Questions were asked about the actual situation, such as hours worked and night and weekend shifts. The response was 53% (n = 411) for internists, 52% (n = 359) for surgeons, and 36% (n = 213) for radiologists. Due to partial non-response, it was unknown for 39 internists, 22 surgeons, and 24 radiologists whether they worked full-time or part-time. They were excluded from the analyses. Furthermore, there was non-response on the dependent variables and consequently another 5 internists, 21 surgeons, and 118 radiologists were excluded. Although radiologists did fill out the questionnaire there were many who did not answer questions on working hours. The remaining 367 internists, 316 surgeons, and 71 radiologists worked in 113, 99, and 51 hospitals respectively. Medical specialists working in academic settings were not included in our survey, because their work arrangements differ markedly from specialists in general hospitals. In academic settings, specialists are employed by the hospital whereas physicians working in general hospitals usually are self-employed and work in partnerships. The Dutch situation is described in Table 1. The internists, surgeons and radiologists included in our sample were compared to all medical specialists in their specialty with respect to age and gender. We did not have all of this information for the same year in which we collected the data, consequently there is lack of up to date information. Only for surgeons and radiologists we have data on gender for all of those we sent a questionnaire. We compared the internists to the population in 1996 and found that the mean age of male internists is about one year higher in our sample. For female internists the mean age is the same in the population of 1996 and our sample. In 1996 83% of the internists were male, while 17% were female, in our sample this is 77% and 23% respectively. For male surgeons the difference in age is just below two years, and female surgeons are about one year older in our sample than in the population of 2001. In that year 94% were male and 6% female, in our sample 90% are male and 10% female. Of the surgeons we sent the questionnaire 92% were male and 8% female. For radiologists we compared our sample to the population of 1997. There is no difference in age for men, for women the difference in age is almost one and a half years. In 1997 93% were male and 7% female; in our sample 79% are male and 21% female. Of the radiologists we sent the questionnaire 85% were male and 15% were female. These differences can be due to demographic shifts. However, based on the non-response analysis, there might have been a slight overrepresentation of female medical specialists in our sample, primarily for the radiologists.

In this article we compared part-time and full-time physicians. We used the following definition of full-time and part-time work, based on self-report by the physicians: 1 Fulltime equivalent (FTE, 100%) is full-time, everything below 1 FTE is part-time. So, 29% of the internists, 18% of the surgeons, and 31% of the radiologists included in our analyses is working part-time. In addition to the question on how many FTE they work they were asked how many hours they work, and how this is divided over several tasks. We analysed this allocation of their working hours over several tasks.

### Multilevel analyses

Medical specialists working in the same hospital are similar concerning hospital characteristics, and, within the same specialty, have similar partnership characteristics. Medical specialists within a partnership most probably agree on the division of tasks. There can be a division of tasks based on skills and interests, or they can equally divide all tasks. Whatever, the number of hours or the proportion of hours spent on different tasks is not independent and this should be taken into account. The data in this study are therefore hierarchical: individual specialists are nested within partnerships, and partnerships are nested within hospitals. Multilevel models are used to analyze hierarchically structured data [22-24]. Multilevel analysis is an extension of ordinary least squares analyses. With ordinary least squares analyses one can estimate, for instance, the relationship between part-time work and hours spent on management tasks in the study population, assuming that there is no partnership or hospital effect in addition to the characteristics of physicians. With multilevel analysis total variation in part-time work is separated into two parts: a part due to differences between physicians, and a part due to differences between partnerships or hospitals. We analysed the specialties separately and this makes the level of the hospital similar to the level of the partnership; the model therefore consists of two levels. We controlled for age and gender in all analyses. Both variables were centred, to give the model interpretable meaning [23].

### Dependent variables

Our dependent variables were all based on self-report: the respondents were asked how many hours they spend on several tasks. With this information proportions of total hours spend on those tasks were computed. As dependent variables we used the total number of hours worked, proportion of hours spent on direct patient care, indirect patient care, and other tasks, and the number of night and weekend shifts per hour worked. Direct patient care consists of: clinical activities and outpatient care for internists; clinical activities, outpatient care, and day treatment for surgeons; outpatient care for radiologists. Indirect patient care is communication and correspondence, and other tasks are education, research, literature, and financial administration. This is the same for all medical specialties in this study. All dependent variables were based on self-report by the medical specialists; they were asked how many hours they spend weekly on certain tasks. Our last dependent variable was the total number of hours worked per FTE. This variable was computed based on self-reports from respondents on the number of hours and what FTE they worked. Differences between part-time and full-time medical specialists were tested with a Wald-test [23].

## Results

The results are presented separately for each specialty.

### Total working hours

Table 2 shows the total number of hours part-time and full-time medical specialists work. Internists and surgeons work on average ten hours per week less in a part-time than in a full-time position. For radiologists the difference between full-time and part-time working hours is eight hours per week.

### Allocation of working hours over tasks

Table 3 shows the allocation of their time over direct patient care, indirect patient care and other tasks. There are no significant differences between full-time and part-time internists, surgeons, and radiologists.

In Table 4 the results of a more in-depth look into their tasks are presented. Proportionally more time is spent on correspondence, but proportionally less time is spent on management tasks by part-time internists (Table 4). No significant differences are found between part-time and full-time surgeons (Table 5). For radiologists we found that part-time working radiologists spend proportionally less time on structural communication (Table 6). Overall we conclude that:

*(1) Part-time medical specialists do not devote proportionally more time to the core business of their work*.

*(2) Part-time medical specialists do not spend proportionally more time on inter-professional communication*.

### Night and weekend shifts

Interestingly, when the number of night and weekend shifts is divided by the total amount of hours worked, assuming that specialists have night and weekend shifts in proportion to their working hours, part-time working internists have proportionally more night shifts than full-time working internists (Table 7). For surgeons and radiologists the differences between full-time and part-time medical specialists are not significant.

*(3) Part-time medical specialists do proportionally no fewer night and weekend shifts than full-time medical specialists*.

### Number of hours worked per FTE

Table 8 shows the number of hours worked per FTE for part-time and full-time working internists, surgeons and radiologists. The only significant difference we found is for surgeons: part-time working surgeons work more hours per FTE than full-time working surgeons. Part-time working internists and radiologists also work more hours per FTE, but these differences are not significant.

*(4) Part-time medical specialists have a similar or higher number of hours worked per FTE*.

## Conclusion and discussion

In this article we examined differences in the allocation of time to direct patient care, indirect patient care, and other tasks between full-time and part-time working internists, surgeons, and radiologists in the Netherlands based on information derived from a questionnaire. We expected part-time medical specialists to spend proportionally more time on direct patient care and inter-professional communication, and less on other tasks. Furthermore, we expected that part-time medical specialists work more hours per FTE.

For none of the medical specialties in this study did we find that part-time medical specialists spend proportionally more time on direct patient care. Part-time internists spend proportionally more time on correspondence and less on management tasks, and part-time radiologists less on structural communication. Except for structural communication for radiologists, these can be considered tasks that are not the core business of medical specialists, but do have potential effects on quality of care. With respect to night and weekend shifts we found that part-time medical specialists do proportionally more or an equal share. It may be that because these shifts are planned in advance, there is less pressure on the non-work-related responsibilities of part-time medical specialists. The number of hours worked per FTE is higher for part-time than for full-time medical specialists, although this difference is only significant for surgeons. In the introduction we related this to efficiency; part-time medical specialists may be less efficient, due to the need for communication, and therefore need more time to do the job. However, there are other explanations for the higher number of hours worked: part-time medical specialists have proportionally more opportunities for investing more hours in their work. The number of hours in a week is limited and full-time medical specialists are closer to the limit than part-time medical specialists.

The idea that part-time medical specialists do less than full-time medical specialists relates primarily to tasks that are not considered core business, 'extra-role' behavior in this article, such as management. It is thought that part-time medical specialists do less, because they may be less devoted to their work [15]. In this article we did not find much evidence to support this idea.

In general, part-time medical specialists do their share of the job. In addition, they work the same or more hours per FTE. However, we only took account of input and did not focus on output. Besides input, output deserves attention as well. Measures of output are numbers of services, quality of care and patient satisfaction. There need not be differences between full-time and part-time medical specialists, but this should still be examined.

Although part-time medical specialists do their share of the job there is still an important consequence of part-time work: more medical specialists are needed to get the work done. Therefore, more medical specialists should be trained. Due to demographic developments and new technologies an increase in demand for health care services can be expected. The increasing number of part-time medical specialists amplifies the need for more medical specialists [5].

In this article our definition of part-time work is working less than 1 FTE. However, there might be a difference between small (say less than 0.5 FTE) and large (say more than 0.9 FTE) part-time jobs. We considered it unnecessary to make a distinction in the size of the part-time job for two reasons. First, amongst medical specialists small part-time jobs are rare; in our data only 10 surgeons, 11 internists, and 8 radiologists work 0.5 FTE or less. Most part-time medical specialists work between 0.5 and 0.9 FTE. Second, analyses in which part-time surgeons were divided into three different groups did not lead to different conclusions from the ones presented in this article.

The hours spent on specialists' tasks, and therefore the allocation of time over different tasks was based on self-report by the medical specialist. These self-reported hours may differ from the actual time spent on certain tasks; medical specialist could over or under-estimate time spent on certain tasks. This over or under-estimation could be related to how they value different tasks. It would be interesting to compare these self-reported data with actual data, to examine whether there are differences. We have information on this subject only for radiologist working in general hospitals in the Netherlands. Van der Velden et al. [25] found that the difference between self-reported and actual working hours was only two percent.

We did not have up-to-date information to test whether our sample is representative of all internists, surgeons and radiologists working in general hospitals in the Netherlands. Differences found by comparing to the available data from some years ago can be due to demographic shifts. However, based on the non-response analysis, there might have been a slight overrepresentation of female medical specialists in our sample, primarily for radiologists. Furthermore, the response for the radiologists was rather low and above that, due to partial non-response, many radiologists were not included in our sample. This might have influenced the results in any direction.

Part-time work in general is sometimes considered to be a typically Dutch phenomenon [26]. Still, there is an international interest in this topic. Increasing numbers of women entering medicine reflect an international trend, and these women work fewer hours per week [12,27,28]. McMurray et al. [12] report that in Australia, Canada, England and the United States between 20% and 50% of all female primary care physicians are working part-time. It is important to study the consequences of the decrease in working hours for the services provided. Increasing demand for health care services can also be found worldwide [28]. Policy should be developed to address these changes in supply and demand. Educating more medical specialists is one possibility, but another is a policy aiming at increasing the contribution of female medical specialists. One example of the latter is to create practice settings that make it possible to better balance work and family life.

In this article we have talked about part-time work as a female concern. Women entering medicine have started the discussion about part-time work. However, we must not forget that nowadays it is not only women who prefer to work part-time; there are also (international) trends for male medical specialists which show a decline in the number of hours worked [2,28,29]. This indicates an overall change in opinion on the number of hours medical specialists should work.

## Competing interests

The author(s) declare that they have no competing interests.

## Authors' contributions

JdJ performed the statistical analyses, drafted the manuscript and contributed to all other aspects of the study. PH contributed to the acquisition of data and was involved in drafting the manuscript. PG participated in the design of the study and the critical revision of the manuscript. LH contributed to the acquisition of data and was involved in drafting the manuscript. All authors have given final approval of the submitted manuscript.

## Pre-publication history

The pre-publication history for this paper can be accessed here:



## Figures and Tables

**Table 1:** Medical specialists in the Netherlands [21]

• Mutual dependency between hospitals and specialists:
Medical specialist can not practice without the hospital
Hospitals can not exist without medical specialists
• Separate payment for hospitals and specialist
• In 2000, 71% of the medical specialists are not in the employ of the hospital
• It is most common for medical specialists to be associated with only one hospital
• Facilities of the hospital are used for outpatient care; outpatient care is part of the hospital

**Table 2:** Hours worked per week (corrected for age and gender), p-value of the difference between full-time and part-time

	Hours worked per week^a^	p-value
***internists***		
part-time	41.64 (1.10)	<0.001
full-time	51.96 (0.75)	
***surgeons***		
part-time	42.96 (1.66)	<0.001
full-time	53.04 (0.58)	
***radiologists***		
part-time	38.15 (3.62)	0.05
full-time	46.33 (1.81)	

^a ^standard error in parentheses

**Table 3:** Proportion of working hours spent on different tasks (corrected for age and gender), with the p-value of the difference between full-time and part-time

	direct patient care^a^	p-value	indirect patient care^a^	p-value	other tasks^a^	p-value
***internists***						
part-time	0.61 (0.02)	0.84	0.19 (0.01)	0.06	0.20 (0.01)	0.27
full-time	0.61 (0.01)		0.17 (0.00)		0.22 (0.01)	
***surgeons***						
part-time	0.71 (0.02)	0.68	0.12 (0.02)	0.89	0.17 (0.01)	0.25
full-time	0.70 (0.01)		0.12 (0.00)		0.18 (0.01)	
***radiologists***						
part-time	0.65 (0.06)	0.89	0.18 (0.06)	0.92	0.17 (0.03)	0.51
full-time	0.66 (0.03)		0.19 (0.03)		0.15 (0.02)	

^a ^standard error in parentheses

**Table 4:** Allocation of total working hours per week over several tasks: internists (corrected for age and gender), p-value of the difference between full-time and part-time

	Mean proportion of hours, part-time^a^	mean proportion of hours, full-time^a^	p-value
clinical activities	0.03 (0.01)	0.04 (0.01)	0.43
outpatient care	0.58 (0.02)	0.57 (0.01)	0.61
Inter-professional communication	0.08 (0.00)	0.08 (0.00)	1
correspondence	0.10 (0.01)	0.08 (0.00)	0.04
education	0.06 (0.01)	0.06 (0.00)	0.78
research	0.02 (0.01)	0.03 (0.00)	0.33
literature	0.06 (0.00)	0.05 (0.00)	0.66
financial administration	0.02 (0.00)	0.01 (0.00)	0.22
management	0.05 (0.01)	0.07 (0.01)	0.03

^a ^standard error in parentheses

**Table 5:** Allocation of total working hours per week over several tasks: surgeons (corrected for age and gender), p-value of the difference between full-time and part-time

	Mean proportion of hours, part-time^a^	mean proportion of hours, full-time^a^	p-value
clinical activities	0.35 (0.02)	0.38 (0.01)	0.28
outpatient care	0.27 (0.01)	0.25 (0.01)	0.31
day care	0.10 (0.01)	0.08 (0.00)	0.22
Inter-professional communication	0.07 (0.01)	0.07 (0.00)	0.86
correspondence	0.05 (0.01)	0.05 (0.00)	0.92
education	0.05 (0.01)	0.04 (0.00)	0.57
research	0.02 (0.00)	0.02 (0.00)	0.34
literature	0.04 (0.00)	0.03 (0.00)	0.42
financial administration	0.01 (0.00)	0.02 (0.00)	0.06
management	0.05 (0.01)	0.06 (0.00)	0.12

^a ^standard error in parentheses

**Table 6:** Allocation of total working hours per week over several tasks: radiologists (corrected for age and gender), p-value of the difference between full-time and part-time

	Mean proportion of hours, part-time^a^	mean proportion of hours, full-time^a^	p-value
outpatient care	0.65 (0.06)	0.66 (0.03)	0.89
structural communication	0.02 (0.03)	0.10 (0.02)	0.04
incidental communication	0.05 (0.01)	0.05 (0.01)	0.92
correspondence	0.05 (0.03)	0.05 (0.02)	1
education	0.03 (0.01)	0.02 (0.01)	0.89
research	0.01 (0.01)	0.01 (0.00)	0.92
literature	0.04 (0.01)	0.04 (0.00)	0.79
financial administration	0.01 (0.01)	0.02 (0.00)	0.89
management	0.07 (0.02)	0.06 (0.01)	0.65

^a ^standard error in parentheses

**Table 7:** Number of night and weekend shifts per hour worked (corrected for age and gender), p-value of the difference between full-time and part-time

	night shifts^a^	p-value	weekend shifts^a^	p-value
***internists***				
part-time	0.43 (0.03)	0.05	1.74 (0.11)	0.07
full-time	0.37 (0.02)		1.49 (0.07)	
***surgeons***				
part-time	0.49 (0.13)	0.26	1.72 (0.58)	0.54
full-time	0.35 (0.02)		1.37 (0.06)	
***radiologists***				
part-time	0.27 (0.07)	0.58	0.91 (0.29)	1.37
full-time	0.31 (0.04)		1.31 (0.16)	

^a ^standard error in parentheses

**Table 8:** Hours worked per FTE (corrected for age and gender), p-value of the difference between full-time and part-time

	Hours worked per FTE^a^	p-value
***internists***		
part-time	58.04 (3.90)	0.15
full-time	52.22 (0.76)	
***surgeons***		
part-time	57.57 (1.77)	0.02
full-time	53.13 (0.58)	
***radiologists***		
part-time	48.63 (3.80)	0.66
full-time	46.76 (1.80)	

^a ^standard error in parentheses
